# Comparing visual-exposure and spatial proximity/coverage metrics of urban blue space in relation to depression-related outcomes: a systematic review and meta-analysis

**DOI:** 10.3389/fpubh.2026.1876283

**Published:** 2026-07-07

**Authors:** Boyi Yang, Yanyan Zhou, Haoyu Liu, Aichun Li

**Affiliations:** 1 School of Physical Education, Hainan Normal University, Haikou, China; 2 School of Agriculture and Environment, University of Western Australia, Perth, WA, Australia; 3 School of Football Education, Hainan Normal University, Haikou, China

**Keywords:** blue space, depression-related outcomes, environmental epidemiology, meta-analysis, spatial proximity/coverage metrics, systematic review, visual-exposure metrics

## Abstract

**Background:**

Urban blue spaces are increasingly examined in relation to mental health, but findings remain difficult to interpret because visual-exposure metrics and spatial proximity/coverage metrics may capture different exposure dimensions. This systematic review and meta-analysis synthesized observational evidence on urban blue-space exposure and depression-related outcomes in adults and compared these two metric categories.

**Methods:**

Following PRISMA 2020, six English and Chinese databases were searched for observational studies published from January 1, 2000, to March 31, 2026. Eligible studies included adults aged 18 years or older and reported depression-related outcomes. Continuous outcomes were synthesized as standardized mean differences using random-effects meta-analysis, and certainty of evidence was assessed using GRADE.

**Results:**

Eight observational studies involving 35,797 adults were included; six studies involving 20,383 participants contributed to the continuous-outcome meta-analysis, whereas two binary-outcome studies were summarized narratively because of differences in outcome ascertainment and exposure modeling. Greater blue-space exposure showed a very small inverse association with depression-related outcomes (SMD = −0.04, 95% CI: −0.06 to −0.02; I^2^ = 38%), and the effect size was trivial at the individual level. By exposure metric type, visual-exposure metrics showed a small inverse but less stable estimate (SMD = −0.07, 95% CI: −0.14 to −0.00; I^2^ = 60%), whereas spatial proximity/coverage metrics showed a similarly small inverse estimate with lower observed heterogeneity (SMD = −0.03, 95% CI: −0.05 to −0.01; I^2^ = 0%). However, each subgroup included only three studies, the subgroup difference was not statistically significant, and the certainty of evidence was very low.

**Conclusions:**

Current observational evidence suggests a trivial inverse association between urban blue-space exposure and depression-related outcomes in adults. Differences between visual-exposure and spatial proximity/coverage metrics should be interpreted descriptively because subgroup differences were not statistically significant and certainty of evidence was very low. These findings may help refine exposure classification but do not support causal or planning-level conclusions.

## 1 Introduction

Urban blue spaces refer to urban environments in which natural or artificial surface water constitutes a defining spatial feature, including rivers, lakes, wetlands, canals, reservoirs, ponds, fountains, and coastal areas (1–3). Rather than originating from a single author or discipline, the term has evolved across ecological planning, sustainable urban development, environmental epidemiology, and urban health research. In earlier planning and design contexts, urban water bodies were mainly discussed as landscape amenities, recreational resources, open-space components, ecological assets, or blue-green infrastructure supporting flood regulation, heat mitigation, biodiversity conservation, and urban resilience. With the increasing integration of sustainability science, public health, and environmental exposure assessment, urban blue spaces have gradually been reconceptualized as measurable environmental exposures that may influence health behavior, psychological restoration, mental health, and population wellbeing (1–3). This conceptual transition reflects a shift from treating urban water bodies mainly as spatial or ecological resources toward examining them as health-relevant environmental determinants embedded in daily urban life.

Depression-related outcomes provide a clinically and public-health-relevant framework through which this environmental exposure can be evaluated. Depression contributes substantially to disease burden, functional impairment, and reduced quality of life, and its occurrence is shaped not only by biological and psychosocial risk factors but also by social, behavioral, and environmental conditions (4). Urban blue spaces may be related to depression-related outcomes through several theoretically grounded pathways. Visual contact with water may support psychological restoration and attentional recovery; spatial proximity or coverage may facilitate walking, recreation, social interaction, and behavioral activation; and blue-space environments may contribute to improved environmental quality and microclimatic regulation in dense urban settings (1, 5–7). These pathways provide a direct rationale for examining blue-space exposure in relation to depression-related outcomes rather than treating blue spaces only as general contributors to subjective wellbeing.

Recent syntheses have suggested generally favorable associations between blue spaces and health, but the evidence remains methodologically fragmented (1–3). Prior reviews have largely synthesized blue-space exposure as a broad environmental category and have focused on overall health, wellbeing, or mental-health outcomes. However, they have not clearly determined whether different exposure operationalizations show comparable associations with depression-related outcomes. This limitation is important because blue-space exposure is not a single construct. Existing studies have used visual-exposure metrics, such as street-view blue proportion or residential visibility indices; spatial proximity/coverage metrics, such as distance to water, buffer-based blue-space coverage, or presence of blue space within a defined area; and, less frequently, subjective or use-based indicators (7–15). These metrics may represent different exposure pathways rather than interchangeable measurements of the same environmental condition.

Several methodological gaps therefore remain unresolved. First, it is unclear whether visual-exposure metrics and spatial proximity/coverage metrics provide similarly consistent evidence for depression-related outcomes. Second, many studies rely on residential exposure measures, which may not capture the locations where individuals spend time or actually experience environmental contact, consistent with the uncertain geographic context problem (16). Third, depression-related outcomes have been measured using heterogeneous instruments, including symptom scales, general mental-health subscales, diagnostic interviews, and self-reported depression or anxiety (8–15). Fourth, the evidence base remains dominated by observational designs, limiting causal inference and leaving reverse causality unresolved. Finally, standardized procedures for exposure classification, effect-size harmonization, and certainty-of-evidence assessment remain insufficiently developed.

The present systematic review and meta-analysis aimed to address these gaps by synthesizing observational evidence on urban blue-space exposure and depression-related outcomes among adults. Two scientific questions guided the review: first, whether greater urban blue-space exposure is associated with lower depression-related outcomes in adults; and second, whether visual-exposure metrics and spatial proximity/coverage metrics differ in the consistency of their associations with these outcomes. The primary objective was to synthesize the overall association between blue-space exposure and adult depression-related outcomes. The secondary objective was to compare, as an exploratory analysis, the consistency of associations across visual-exposure and spatial proximity/coverage metric categories. Conceptually, visual-exposure metrics may be more closely aligned with potential restorative visual contact, whereas spatial proximity/coverage metrics may better approximate spatial opportunities for recreation, physical activity, social interaction, and behavioral activation. These metric categories therefore require separate interpretation and should not be treated as interchangeable measures of the same exposure pathway.

We hypothesized that greater blue-space exposure would be associated with slightly lower depression-related outcomes. We further hypothesized, in an exploratory manner, that spatial proximity/coverage metrics would show lower observed heterogeneity than visual-exposure metrics. Because of the small number of studies and the heterogeneity of outcome instruments, these hypotheses were intended to guide evidence synthesis rather than to support causal or planning-level inference.

## 2 Materials and methods

This study followed the PRISMA 2020 guidelines (17) and was registered with PROSPERO (CRD420261340412). Registration URL: https://www.crd.york.ac.uk/PROSPERO/view/CRD420261340412.

### 2.1 Data sources and search strategy

We systematically searched Web of Science, PubMed, Scopus, American Psychological Association PsycInfo, China National Knowledge Infrastructure, and Wanfang for studies published between January 1, 2000, and March 31, 2026. The search combined terms related to blue spaces, adult populations, and depressive symptoms. These databases were selected to cover biomedical, psychological, environmental-health, urban-planning, and Chinese-language literature. The year 2000 was selected because geographic information system-based environmental exposure assessment and urban environmental epidemiology became more widely used after this period, making earlier exposure metrics less comparable with contemporary blue-space studies. Full database-specific search strategies, including Boolean operators, field tags, date limits, and language restrictions, are provided in Supplementary Table S1.

### 2.2 Inclusion and exclusion criteria

**Figure 1:**
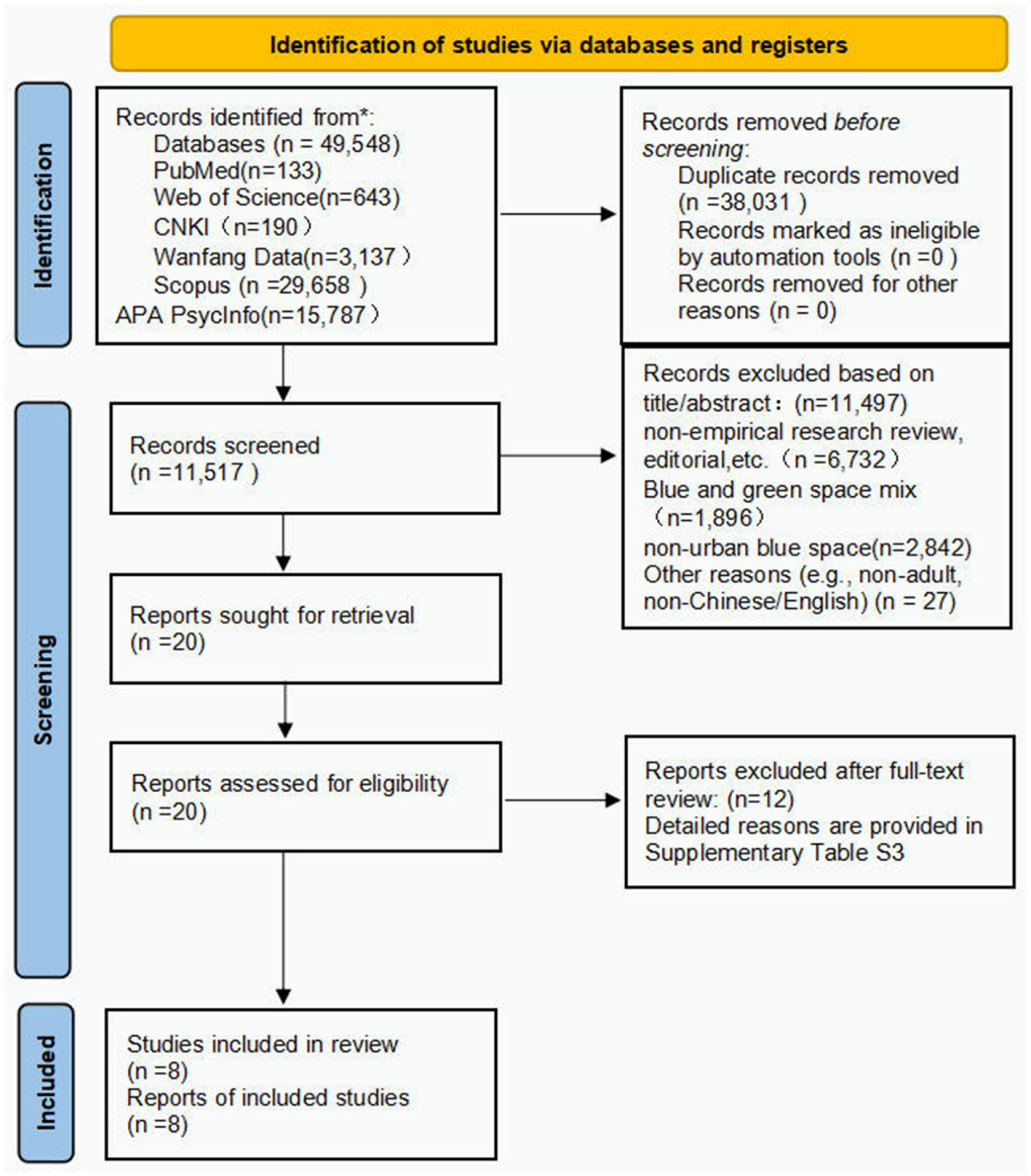
Flowchart of literature screening process.

The eligibility criteria were defined using the Population, Intervention or exposure, Comparison, Outcomes, and Study design framework. Studies were eligible if they included adults aged 18 years or older; assessed exposure to urban blue spaces, including rivers, lakes, coastal areas, wetlands, or related water bodies; reported depression-related outcomes, including symptom scales, general psychological distress scales, mental-health subscales, diagnostic mood-disorder outcomes, antidepressant use, or self-reported depression/anxiety outcomes; used a cross-sectional, cohort, or case-control design; and provided full text in English or Chinese. Studies were excluded if they assessed green space only, reported combined green-blue exposure without a separable blue-space estimate, focused only on artificial indoor water features, reported non-depression-specific wellbeing outcomes, or were reviews, conference abstracts, duplicate publications, or animal studies. Children and adolescents were excluded because their activity spaces, school-based exposures, developmental characteristics, and dependence on parental mobility may differ substantially from adults. Studies assessing combined green-blue exposure were included only when the blue-space effect estimate was reported separately or could be extracted independently. Two reviewers independently screened titles, abstracts, and full texts, with disagreements resolved through discussion or consultation with a third reviewer. The study-selection process is shown in Figure 1. Full-text exclusion reasons are provided in Supplementary Table S3.

### 2.3 Data extraction

Two researchers independently extracted study-level aggregate data for the meta-analysis, including first author, year of publication, country, study design, sample size, mean age of participants, blue-space exposure metric, depression-related outcome measure, and reported effect estimate with its 95% confidence interval. All extracted information was obtained from published articles and their supplementary materials.

### 2.4 Exposure classification

Blue-space exposure metrics were classified into two pre-specified categories. Visual-exposure metrics referred to measures intended to quantify visible contact with blue spaces, including street-view blue proportion and residential visibility indices. Spatial proximity/coverage metrics referred to geographic measures indicating residential proximity to, presence of, or amount of blue space, including distance to blue space, blue-space coverage within residential buffers, and presence or absence of blue space within a defined area. These metrics were interpreted as spatial proximity or coverage indicators and were not assumed to measure actual physical access, public entry, use, or exposure duration. These categories were treated as methodological exposure groupings rather than equivalent causal mechanisms.

### 2.5 Quality assessment

Methodological quality was assessed using the Downs and Black checklist (18). This tool was used to evaluate reporting quality, confounding control, exposure measurement, outcome measurement, statistical testing, and sample representativeness across non-randomized studies. Each item was scored from 0 to 2, yielding a total score of 0–16. Scores were interpreted descriptively as excellent (≥13), good (10–12), fair (7–9), or poor ( ≤ 6). These summary scores were used to support methodological interpretation rather than as evidence of low risk of bias. Full-text exclusion decisions were based on eligibility criteria, extractability of relevant effect estimates, and methodological adequacy for synthesis, with detailed reasons reported in Supplementary Table S3. Because summary quality scores may not fully capture domain-specific bias in observational environmental epidemiology, concerns related to confounding, exposure misclassification, reverse causality, and indirectness were considered qualitatively in the Grading of Recommendations Assessment, Development and Evaluation assessment and in the limitations. To complement the Downs and Black summary scores, domain-based risk-of-bias considerations focusing on study design, residual confounding, green-space confounding, exposure misclassification, outcome measurement, and reverse causality are provided in Supplementary Table S5.

### 2.6 Statistical analysis

RevMan 5.4 was used for the meta-analysis. Continuous depression-related outcomes were synthesized as standardized mean differences to improve comparability across studies using different depression-related instruments. Effect directions were standardized so that negative values indicated lower depression-related outcomes among participants with greater blue-space exposure. When studies reported adjusted regression coefficients, coefficients were standardized using the study-specific standard deviation of the depression-related outcome. Standard errors were derived from reported confidence intervals when necessary. Because exposure contrasts differed across studies, the pooled standardized mean difference was interpreted as a standardized comparison of higher vs. lower blue-space exposure rather than as a dose–response estimate. The original effect estimates, exposure contrasts, scale directions, transformation rules, final standardized mean differences, and standard errors are provided in Supplementary Table S2. For studies reporting group contrasts, standardized mean differences were calculated as the difference in depression-related outcome scores between higher and lower blue-space exposure groups divided by the pooled outcome standard deviation. For studies reporting adjusted regression coefficients, coefficients were converted to standardized mean differences by multiplying the reported exposure contrast or increment by the regression coefficient and dividing by the study-specific standard deviation of the depression-related outcome. Standard errors were derived from reported 95% confidence intervals using SE = (upper limit – lower limit) / (2 × 1.96) when necessary. Outcome standard deviations were extracted from the original articles or supplementary materials when available; when not directly reported, the derivation source and assumptions were documented in Supplementary Table S2. These conversions assumed that the reported exposure contrast represented a higher-vs.-lower blue-space comparison and that the outcome standard deviation was an appropriate scale-standardizing denominator. A score-based or ordinal dose-response synthesis was considered but was not conducted because the included studies used non-comparable exposure contrasts, including quartile comparisons, binary presence/absence indicators, distance categories, buffer-based coverage, and continuous percentage or area increments. These contrasts could not be converted into a common exposure dose without imposing assumptions that were not supported by the original studies. Therefore, the synthesis was restricted to standardized higher-vs.-lower exposure comparisons and interpreted directionally rather than as a dose-response estimate.

Binary depression-related outcomes were not pooled because only two studies reported such outcomes and they differed in outcome ascertainment, exposure scaling, and model specification. These studies were therefore summarized narratively and tabulated separately.

Heterogeneity was assessed using the I^2^ statistic, with values below 25%, 25%−50%, and above 50% interpreted as low, moderate, and substantial heterogeneity, respectively (19). Random-effects models using the DerSimonian–Laird method were applied because clinical and methodological heterogeneity was expected (20). Because the number of studies contributing to the primary meta-analysis was small, an additional Hartung–Knapp sensitivity analysis was conducted based on the extracted standardized mean differences and standard errors to examine whether the inference was robust to small-sample random-effects assumptions. The primary quantitative synthesis included the overall continuous-outcome meta-analysis and the pre-specified comparison between visual-exposure metrics and spatial proximity/coverage metrics. A test for subgroup differences was conducted in RevMan and interpreted cautiously because only three studies were available in each exposure-metric category.

A sensitivity meta-analysis restricted to strictly depression-specific continuous instruments was considered but was not statistically feasible because only one continuous-outcome study used a depression-specific symptom scale. Therefore, the main synthesis was interpreted as a synthesis of depression-related outcomes rather than strictly depressive symptoms. Given the small number of studies contributing to the primary meta-analysis, pooled estimates were interpreted cautiously and supported by leave-one-out sensitivity analysis and certainty-of-evidence assessment. Funnel plots were inspected only exploratorily because fewer than 10 studies were available; formal asymmetry testing was not performed, and publication bias could not be ruled out.

### 2.7 Certainty of evidence assessment

The certainty of evidence for the visual-exposure and spatial proximity/coverage subgroups was assessed using the GRADE framework (21, 22). Because the included studies were observational, the starting certainty was rated as low. Certainty was then downgraded, where appropriate, across five domains: risk of bias, inconsistency, indirectness, imprecision, and publication bias. Inconsistency was judged using I^2^, overlap of confidence intervals, and sensitivity of subgroup estimates to influential studies. Imprecision was judged according to the number of contributing studies, width of confidence intervals, and proximity of estimates to the null. Publication bias was judged cautiously because fewer than 10 studies were available for quantitative synthesis. Two reviewers independently performed the assessments, with disagreements resolved through discussion. Domain-level judgments and reasons for downgrading are reported in the GRADE summary-of-findings table.

## 3 Results

### 3.1 Literature search and study characteristics

The initial search yielded 49,548 records. After duplicate removal, 11,517 records remained for title and abstract screening, of which 11,497 were excluded. Twenty full-text reports with traceable bibliographic records were assessed for eligibility. Twelve reports were excluded after full-text review because of incompatible outcomes, inseparable or incompatible exposure estimates, insufficient extractable effect-size data, ineligible populations, or lack of eligible primary effect-size data. Detailed exclusion reasons are provided in Supplementary Table S3 and summarized in Figure 1. Eight observational studies (8–15) were included in the review, of which six contributed to the meta-analysis of continuous depression-related outcomes and two reported binary depression-related outcomes that were summarized narratively. Included studies were conducted in China, the United Kingdom, Spain, the Netherlands, and New Zealand; seven were cross-sectional and one was longitudinal. Downs and Black summary scores ranged from 13 to 16. These scores indicated generally adequate reporting and methodological transparency, but they were not interpreted as evidence of low risk of bias. Because most included studies were observational and cross-sectional, the summary scores were interpreted alongside domain-based risk-of-bias considerations, including residual confounding, exposure misclassification, green-space confounding, and reverse causality. Study-specific characteristics are presented in Table 1, and dimension-specific quality scores are presented in Table 2.

**Table 1:** Characteristics of included studies.

No.	References	Country	Design	Sample size	Mean age	Blue space exposure metric	Depression outcome
1	Helbich et al, (8)	China	Cross-sectional	1,190	71	Street-view blue proportion (Q4 vs. Q1)	GDS-15
2	White et al, (9)	UK	Longitudinal	15,361	45	Coastal distance ( ≤ 5 km vs. 45-50 km)	GHQ-12
3	Liu et al, (10)	China	Cross-sectional	1,274	40	Water area within 300 m (per ha)	MHI-5
4	Chen et al, (11)	China	Cross-sectional	966	70	Water proximity index (1 vs. 0)	SF-36 MH
5	Nutsford et al, (12)	New Zealand	Cross-sectional	442	45	Visible blue space (per 10% increase)	K10
6	Liu et al, (13)	China	Cross-sectional	1,150	40	Street-view blue proportion (per 1%)	GHQ-12
7	Triguero-Mas, 2015 (14)	Spain	Cross-sectional	8,793	41	Blue space within 300 m (yes/no)	Self-reported depression/anxiety
8	de Vries et al, (15)	Netherlands	Cross-sectional	6,621	45	Blue space coverage within 1 km (per 1%)	CIDI mood disorder

**Table 2:** Quality assessment scores (downs & black tool, max 16 points).

No.	Study	Study design	Confounding control	Exposure measurement	Outcome measurement	Results reporting	Statistical tests	Sample representativeness	Follow-up duration	Total
1	Helbich et al, (8)	2	2	2	2	2	2	1	0	13
2	White et al, (9)	2	2	2	2	2	2	2	2	16
3	Liu et al, (10)	2	2	2	2	2	2	1	0	13
4	Chen et al, (11)	2	2	2	2	2	2	1	0	13
5	Nutsford et al, (12)	2	2	2	2	2	2	2	0	14
6	Liu et al, (13)	2	2	2	2	2	2	1	0	13
7	Triguero-Mas et al, (14)	2	2	1	2	2	2	2	0	13
8	de Vries et al, (15)	2	2	2	2	2	2	2	0	14

Scoring based on Downs & Black (1998) tool, total 16 points. Score ≥13 = excellent, 10–12 = good, 7–9 = fair, ≤ 6 = poor. These summary scores reflect reporting and methodological quality and were not interpreted as equivalent to low risk of bias; domain-based risk-of-bias considerations are provided in Supplementary Table S5.

### 3.2 Exposure and outcome measurement

Blue-space exposure metrics were grouped into visual-exposure metrics and spatial proximity/coverage metrics for the pre-specified exposure-metric comparison. These categories reflected study-level operational definitions of exposure metrics rather than harmonized individual-level exposure measurements. Visual-exposure metrics included street-view blue proportion and residential visibility indices. Spatial proximity/coverage metrics included geographic information system-calculated distance, blue-space area or coverage within buffers, and presence or absence of blue space within a defined area. Depression-related outcomes included GDS-15, GHQ-12, MHI-5, SF-36 mental health scores, K10, CIDI-based mood disorder, and self-reported depression/anxiety. These instruments were further classified as depression-specific symptom scales, general psychological distress scales, mental-health subscales, diagnostic mood-disorder outcomes, and self-reported depression/anxiety outcomes. Because only **one** continuous-outcome study used a strictly depression-specific symptom scale, a depression-specific-only meta-analysis was not performed. The **two** binary-outcome studies were not pooled with the continuous-outcome studies because they differed in outcome ascertainment, exposure scaling, and model specification; they are therefore presented narratively and in a separate table.

### 3.3 Continuous-outcome meta-analysis and exposure-metric comparison

Six studies contributed to the meta-analysis of continuous depression-related outcomes. The pooled association was statistically significant but very small in magnitude (SMD = −0.04, 95% CI: −0.06 to −0.02; *p* < 0.0001). Heterogeneity was low to moderate (I^2^ = 38%, *p* = 0.15). Given the small effect size and observational evidence base, this estimate should not be interpreted as evidence of a clinically meaningful individual-level effect.

**Figure 2:**
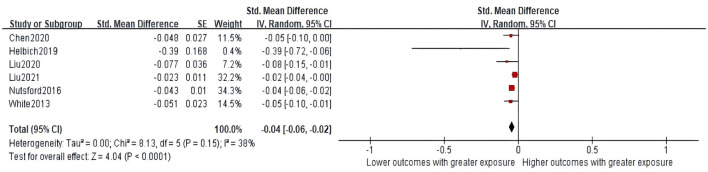
Forest plot for continuous outcomes. Study-level standardized mean differences were synthesized using a DerSimonian–Laird random-effects model with inverse-variance weighting. Negative values indicate lower depression-related outcomes among participants with greater blue-space exposure. Horizontal lines represent 95% confidence intervals, and the diamond represents the pooled estimate.

At the individual-study level, the direction-standardized estimates generally pointed toward lower depression-related outcomes among participants with greater blue-space exposure, but the magnitude and precision of estimates varied across studies. Helbich 2019 showed the largest standardized estimate and contributed to heterogeneity in the visual-exposure subgroup. Individual study estimates are shown in Figure 2, and the effect-size conversions are detailed in Supplementary Table S2.

**Figure 3:**
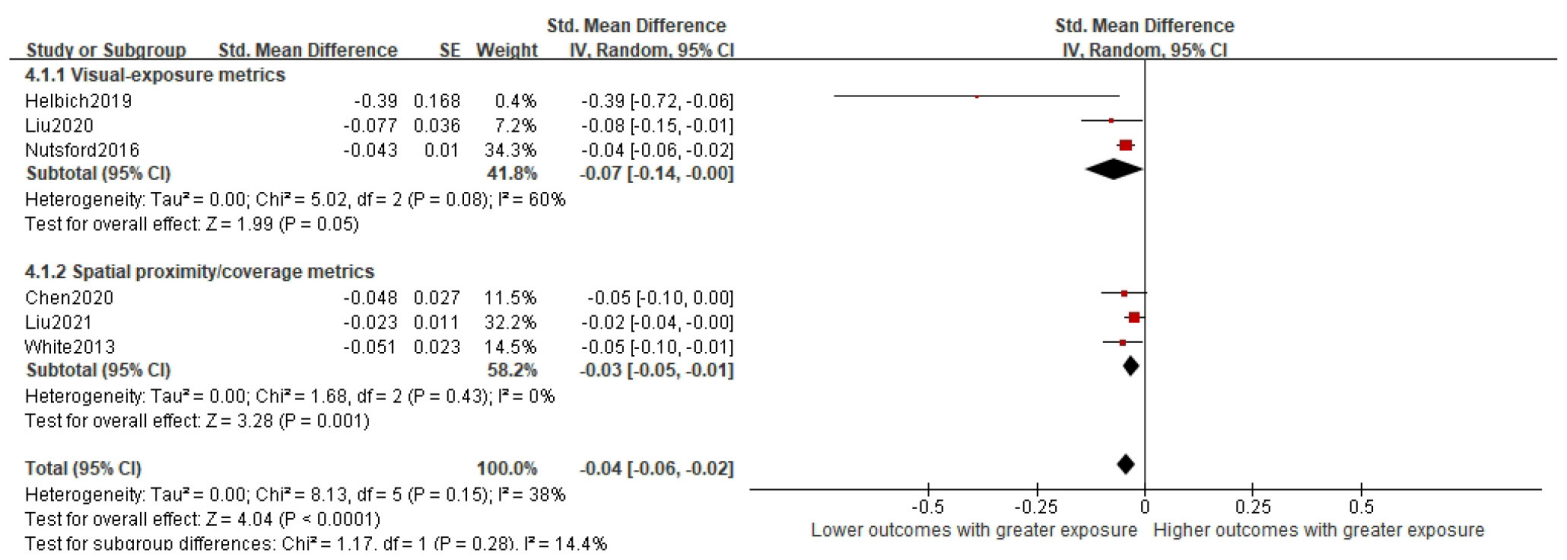
Subgroup analysis by exposure type. Exploratory subgroup analysis by exposure metric type. Study-level standardized mean differences were pooled using a DerSimonian–Laird random-effects model with inverse-variance weighting. Negative values indicate lower depression-related outcomes among participants with greater blue-space exposure. The test for subgroup differences was interpreted cautiously because each subgroup included only three studies.

Because the title and research question focus on exposure-metric classification, the comparison between visual-exposure metrics and spatial proximity/coverage metrics was treated as a main analytical component. For visual-exposure metrics, the pooled SMD was −0.07 (95% CI: −0.14 to −0.00; I^2^ = 60%). For spatial proximity/coverage metrics, the pooled SMD was −0.03 (95% CI: −0.05 to −0.01; I^2^ = 0%). The exploratory test for subgroup differences did not show a statistically significant difference between the two metric categories (χ^2^ = 1.17, df = 1, p = 0.28, I^2^ = 14.4%). Therefore, the observed difference in heterogeneity was interpreted descriptively rather than as evidence of a statistically confirmed subgroup effect. The subgroup analysis by exposure metric type is shown in Figure 3.

### 3.4 Binary outcomes

Two studies reported binary depression-related outcomes and were summarized narratively. Triguero-Mas 2015 (14) reported a non-significant association between blue space within 300 m and self-reported depression/anxiety (OR = 1.13, 95% CI: 0.90–1.41). In contrast, de Vries 2016 (15) reported lower odds of CIDI-based mood disorder with greater blue-space coverage within 1 km (OR = 0.97, 95% CI: 0.951–0.989). Because these two studies differed in outcome ascertainment, exposure scaling, and model specification, they were not statistically pooled. Their findings are presented in Table 3 and considered separately from the main continuous-outcome meta-analysis.

**Table 3:** Binary depression-related outcomes summarized narratively.

Study	Country	Sample size	Outcome ascertainment	Exposure metric	Effect estimate	Interpretation	Reason not statistically pooled
Triguero-Mas 2015	Spain	8,793	Self-reported depression/anxiety	Presence or absence of blue space within 300 m	OR = 1.13 (95% CI: 0.90–1.41)	The estimate was not statistically significant; OR > 1 suggests higher odds but the CI includes the null.	Not pooled because the outcome was self-reported depression/anxiety and the exposure was binary presence/absence, which differed from the CIDI-based mood-disorder outcome and continuous blue-space coverage metric used in de Vries 2016
de Vries 2016	Netherlands	6,621	CIDI-based mood disorder	Blue-space coverage within 1 km; per 1% increase	OR = 0.97 (95% CI: 0.951–0.989)	OR < 1 suggests lower odds of mood disorder with greater blue-space coverage.	Not pooled because the outcome was a diagnostic interview-based mood disorder and the exposure was modeled as continuous coverage, which differed from the binary self-reported outcome and binary exposure metric used in Triguero-Mas 2015

### 3.5 Additional exploratory analyses

Exploratory subgroup analyses were conducted by exposure metric type and blue-space type. The exposure-metric comparison is presented as part of the main continuous-outcome results because it directly corresponds to the revised title and research question. The exploratory subgroup analysis by blue-space type was moved to the Supplementary Materials because the inland and coastal/mixed categories each included only three studies and combined different exposure-metric types. Therefore, this analysis was interpreted only as a supplementary descriptive check and was not used to inform the main interpretation.

### 3.6 Sensitivity analysis and publication bias

**Figure 4:**
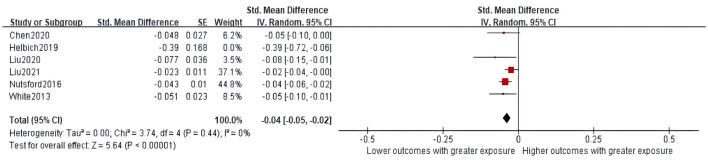
The displayed recalculated estimate shows the result after excluding Helbich 2019, the influential study identified in the sensitivity analysis. Estimates were synthesized using a DerSimonian–Laird random-effects model. Negative values indicate lower depression-related outcomes among participants with greater blue-space exposure. Horizontal lines represent 95% confidence intervals, and the diamond represents the pooled estimate.

Leave-one-out sensitivity analyses showed that the overall pooled estimate remained similar in magnitude after sequential removal of each continuous-outcome study, with pooled standardized mean differences ranging from −0.045 to −0.038. When Helbich 2019 was removed, the pooled estimate remained similar in magnitude, but heterogeneity decreased from I^2^ = 38% to I^2^ = 0%. The corresponding sensitivity analysis is shown in Figure 4. Additional sensitivity analyses were conducted to examine the robustness of the pooled estimate under different analytical assumptions. The Hartung–Knapp sensitivity analysis produced a similar pooled point estimate but a wider confidence interval than the primary DerSimonian–Laird model (SMD = −0.04, 95% CI: −0.07 to −0.01), indicating that the direction of association was broadly consistent but statistical precision remained limited under small-sample random-effects assumptions. After excluding older-adult samples, the pooled estimate remained very small (SMD = −0.04, 95% CI −0.05 to −0.02; I^2^ = 16%). Because only one longitudinal study was included, formal subgroup meta-analysis by study design was not feasible. A cross-sectional-only sensitivity analysis yielded a similarly very small pooled estimate (SMD = −0.04, 95% CI −0.07 to −0.02; I^2^ = 49%). The only longitudinal study, White 2013, also reported a small inverse association (SMD = −0.05, 95% CI −0.10 to −0.01). Therefore, the overall pooled estimate should be interpreted as being driven primarily by cross-sectional evidence. Detailed sensitivity-analysis results, including exposure-metric-specific leave-one-out analyses, are provided in Supplementary Table S6. Exposure-metric-specific leave-one-out analyses were conducted exploratorily because each exposure-metric subgroup included only three studies, and removal of one study left only two studies per recalculated estimate. These analyses were therefore interpreted descriptively rather than as confirmatory sensitivity analyses. Overall, the spatial proximity/coverage subgroup remained small in magnitude and directionally inverse across recalculations, whereas the visual-exposure subgroup showed greater instability across one-study exclusions. These findings further support the cautious interpretation of exposure-metric differences. Funnel plots were inspected only exploratorily because only six studies contributed to the continuous-outcome meta-analysis. Publication bias and small-study effects could not be ruled out.

### 3.7 GRADE certainty of evidence

The certainty of evidence was rated as very low for both exposure categories. Risk of bias was rated as serious because most included studies were cross-sectional, exposure assessment was heterogeneous and largely residential, and residual confounding by socioeconomic conditions, green space, urban density, and environmental quality could not be excluded. For visual-exposure metrics, certainty was further downgraded because of serious inconsistency, serious imprecision, and suspected publication bias (21, 22). For spatial proximity/coverage metrics, certainty was further downgraded because of serious imprecision and suspected publication bias. Although no observed heterogeneity was detected in this subgroup, the estimate was based on only three studies. Therefore, the true effect for both exposure categories may be substantially different from the current estimates. The GRADE summary of findings is presented in Table 4.

**Table 4:** GRADE summary of findings: visual-exposure and spatial proximity/coverage metrics in relation to depression-related outcomes in adults.

Outcome	No. of studies (participants)	Study design	Risk of bias	Inconsistency	Indirectness	Imprecision	Publication bias	Effect estimate SMD [95% CI]	Certainty
Depression-related outcomes (overall blue space exposure)	6 (20,383)	Observational	Serious	Not serious (I^2^ = 38%)	Not serious	Serious^∧^a^∧^	Could not be ruled out^∧^b^∧^	−0.04 [−0.06, −0.02]	⊗○○○ VERY LOW
Depression-related outcomes (visual-exposure metrics subgroup)	3 (2,782)	Observational	Serious	Serious^∧^c∧ (I^2^ = 60%)	Not serious	Serious∧a∧	Could not be ruled out^∧^b^∧^	−0.07 [−0.14, 0.00]	⊗○○○ VERY LOW
Depression-related outcomes (spatial proximity/coverage metrics subgroup)	3 (17,601)	Observational	Serious	Not serious (I^2^ = 0%)	Not serious	Serious^∧^a^∧^	Could not be ruled out^∧^b^∧^	−0.03 [−0.05, −0.01]	⊗○○○ VERY LOW

Because all included studies were observational, the starting certainty was rated as low. a. Imprecision was rated as serious because each subgroup included only three studies, confidence intervals were close to the null, and the pooled effects were very small in magnitude. b. Publication bias could not be reliably assessed because fewer than 10 studies were available for quantitative synthesis; funnel plot interpretation was exploratory and Egger's test was not performed. Therefore, potential publication bias or small-study effects could not be ruled out. c. Inconsistency was rated as serious for the visual-exposure subgroup because moderate heterogeneity was observed (I^2^ = 60%) and the subgroup estimate was sensitive to one influential study. After removing Helbich et al., heterogeneity decreased, indicating that this study contributed substantially to between-study variability. d. Although no observed heterogeneity was detected for spatial proximity/coverage metrics, this judgment should be interpreted cautiously because the subgroup included only three studies. e. Risk of bias was rated as serious because most included studies were cross-sectional, exposure assessment was heterogeneous and largely residential, and residual confounding by socioeconomic conditions, green space, urban density, and environmental quality could not be excluded.

## 4 Discussion

### 4.1 Principal findings

This systematic review and meta-analysis found a very small inverse association between urban blue-space exposure and depression-related outcomes among adults. Exploratory subgroup analyses suggested lower observed heterogeneity for spatial proximity/coverage metrics than for visual-exposure metrics. However, these subgroup findings were based on only three studies per category, and the certainty of evidence was rated as very low. Therefore, the findings should be interpreted as preliminary and hypothesis-generating rather than as confirmatory evidence for the superiority of one exposure pathway. The first hypothesis, that greater blue-space exposure would be associated with slightly lower depression-related outcomes, was supported only in the sense of a very small inverse association. The second hypothesis, that spatial proximity/coverage metrics would show lower observed heterogeneity than visual-exposure metrics, was descriptively supported, but the subgroup difference was not statistically significant and each subgroup included only three studies. Therefore, the comparison should be interpreted as evidence of measurement-level differences in current studies rather than as confirmation of different causal pathways.

Interpreted through the lens of integrative sustainability science (23), the observed association—albeit marginal—may represent a potential health co-benefit of blue infrastructure that is primarily justified for flood control, heat mitigation, and biodiversity conservation.

### 4.2 Comparison with prior evidence and interpretation of effect magnitude

The pooled SMD of −0.04 is substantially smaller than estimates reported in previous meta-analyses of mixed-age populations (SMD ≈ −0.25 to −0.35) (2), though it aligns more closely with adult-specific estimates (3, 24). Several factors may partly explain this discrepancy, although these explanations were not directly tested in the included studies. First, our analysis was restricted to adults, a population with more dispersed activity spaces and consequently greater exposure misclassification when residential-based metrics are used (16). Second, we excluded studies reporting only general wellbeing outcomes without depression-specific thresholds, yielding more conservative estimates. Third, strict eligibility and extractability criteria narrowed the evidence base. Several studies were excluded because they did not provide separable blue-space estimates, reported combined green-blue exposure, included ineligible populations, or did not report compatible depression-related outcomes. Therefore, the smaller pooled estimate may partly reflect a more restrictive synthesis rather than a direct contradiction of broader reviews.

The pooled effect size should be interpreted cautiously. Although the association was statistically significant, its magnitude was very small and should not be interpreted as evidence of a clinically meaningful individual-level effect. At the population level, such an association may be relevant only if it is causal, broadly distributed, and achieved through infrastructure already justified by ecological, recreational, or climate-adaptation objectives. Therefore, the present findings do not support blue-space access as a standalone mental-health intervention. Rather, they suggest that mental-health benefits may be considered possible co-benefits when blue-space access is incorporated into projects already justified by climate adaptation, recreation, flood management, or ecological restoration. This interpretation remains provisional and requires confirmation in longitudinal, quasi-experimental, or intervention studies.

### 4.3 Heterogeneity and exposure pathways

The observed difference in heterogeneity between spatial proximity/coverage metrics and visual-exposure metrics may reflect measurement differences rather than distinct causal pathways. Spatial proximity/coverage indicators, such as distance to blue space or residential buffer coverage, are relatively stable geographic measures, but they do not necessarily capture actual use, exposure duration, perceived accessibility, or environmental quality. This limitation is consistent with the uncertain geographic context problem, whereby residential neighborhoods may not reflect where individuals actually experience environmental exposures (16). Therefore, proximity should not be interpreted as equivalent to meaningful exposure (7).

Visual-exposure metrics, such as street-view blue proportion and residential visibility indices, may capture potential visual contact with water but are more context-dependent. Their relevance may vary by perceived safety, water quality, surrounding built form, weather conditions, and individual receptivity, consistent with restoration theory and ecosystem-service perspectives (5, 6). The larger heterogeneity in this subgroup should therefore be interpreted as measurement and contextual variability rather than evidence that visual exposure is unimportant. Moreover, the visual-exposure subgroup was sensitive to Helbich et al. (8), whose removal reduced heterogeneity, suggesting that this study contributed substantially to the instability of the visual-exposure subgroup estimate. Future studies should distinguish visibility, proximity, accessibility, perceived quality, actual use, and activity-space exposure, and further examine the role of urban morphology (16, 25).

### 4.4 Limitations and certainty of evidence

Differences in covariate adjustment, including control for socioeconomic factors, green-space exposure, air pollution, and built-environment characteristics, are summarized in Supplementary Table S4. Several limitations should be considered. First, seven of the eight included studies were cross-sectional, which precludes causal inference and leaves reverse causality unresolved. Second, exposure assessment was heterogeneous and largely based on residential metrics, making the findings vulnerable to exposure misclassification and the uncertain geographic context problem (16). Third, the evidence base was small, publication bias could not be reliably assessed, and subgroup analyses included only three studies per exposure category. Fourth, the included studies were conducted in a limited number of national contexts, including China, the United Kingdom, Spain, the Netherlands, and New Zealand, which restricts generalizability to low-income settings, tropical cities, and other underrepresented urban contexts. In addition, the exclusion of studies with combined green-blue estimates, non-adult or mixed-age populations, or non-separable exposure contrasts highlights a broader limitation of the current evidence base. The lack of standardized exposure contrasts across studies also prevented dose-response synthesis, including score-based or ordinal high-vs.-low comparisons. Future blue-space studies should report harmonized exposure categories, separable blue-space estimates, and standardized depression-related outcomes to enable more robust meta-analysis. Accordingly, the certainty of evidence was rated as very low for both visual-exposure metrics and spatial proximity/coverage metrics. These findings should therefore be interpreted as preliminary associations rather than definitive evidence for association or planning-level recommendations.

### 4.5 Implications for future research and exposure assessment

From a future research and exposure-assessment perspective, the findings highlight the need to distinguish different exposure dimensions, including physical access, proximity, visibility, perceived quality, actual use, and exposure duration. Visual-exposure metrics may be more relevant to design questions concerning visibility, sightlines, street-level visual contact, and perceived blue-space presence, whereas spatial proximity/coverage metrics may be more relevant to walkable access, spatial availability, public entry points, and connectivity to blue spaces. However, these metrics should not be treated as direct measures of actual use, restorative experience, or causal exposure pathways. Broader climate-smart public-health and climate-adaptation literature likewise emphasizes context-specific evaluation of the potential health co-benefits and harms of environmental interventions (29, 30).

The finding that spatial proximity/coverage metrics showed lower observed heterogeneity should therefore be interpreted as suggesting a more consistent measurement category within the limited current evidence base, not as evidence that spatial proximity/coverage indicators are superior to visual-exposure indicators. Similarly, the instability of the visual-exposure subgroup should not be interpreted as evidence that visual exposure is unimportant. Rather, these findings indicate that future blue-space health research should distinguish exposure visibility, spatial proximity, accessibility, perceived quality, actual use, and activity-space exposure before drawing conclusions about which pathway is most relevant for depression-related outcomes.

Equity considerations remain important because blue-space access, visibility, quality, and use may be unevenly distributed across socioeconomic groups. Access improvements may also contribute to blue or green gentrification if they are not accompanied by safeguards against displacement (26, 27). Therefore, access-oriented planning should be evaluated alongside housing affordability, community participation, and distributional equity.

Future studies should prioritize longitudinal, quasi-experimental, and intervention designs with repeated activity-space-based exposure assessment to address reverse causality and the uncertain geographic context problem (16). Standardized depression measures, transparent effect-size conversion, and harmonized exposure contrasts are needed to improve comparability across studies. Future evidence syntheses should continue to apply transparent procedures for effect-size standardization and random-effects meta-analysis (31). Future work should also examine potential mediators, including psychological restoration, physical activity, social interaction, behavioral activation, and microclimatic regulation (5, 6, 28). Such work can be situated within wider sustainable-development, climate-risk, population-prevention, health-equity, and Nature-based Solutions frameworks (32–36).

## 5 Conclusions

This systematic review and meta-analysis found a very small inverse association between urban blue-space exposure and depression-related outcomes among adults. Exploratory subgroup analyses suggested that spatial proximity/coverage metrics showed lower observed heterogeneity than visual-exposure metrics, but this finding was based on few studies and very low-certainty evidence.

The results should therefore not be interpreted as definitive evidence that spatial proximity/coverage indicators are superior to visual-exposure indicators, or that blue-space exposure can serve as a standalone mental-health intervention. Rather, the findings highlight the need for more rigorous longitudinal and quasi-experimental studies using standardized depression measures, activity-space-based exposure assessment, and clearer distinctions between different forms of blue-space exposure. Pending stronger causal evidence, these findings should primarily be used to generate hypotheses and improve exposure assessment in future blue-space health research rather than to guide planning interventions.
