# Supplementary figures and images for: Comparing visual-exposure and spatial proximity/coverage metrics of urban blue space in relation to depression-related outcomes: a systematic review and meta-analysis
Source: Front Public Health. 2026 Jul 7;14:1876283. doi: 10.3389/fpubh.2026.1876283 (PMC13384931; doi:10.3389/fpubh.2026.1876283)

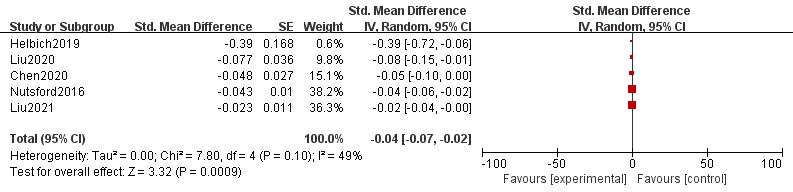

Supplement: Supplementary file 7 [file Image_1.png]

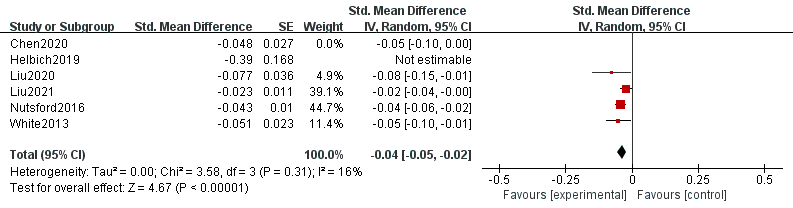

Supplement: Supplementary file 8 [file Image_2.png]

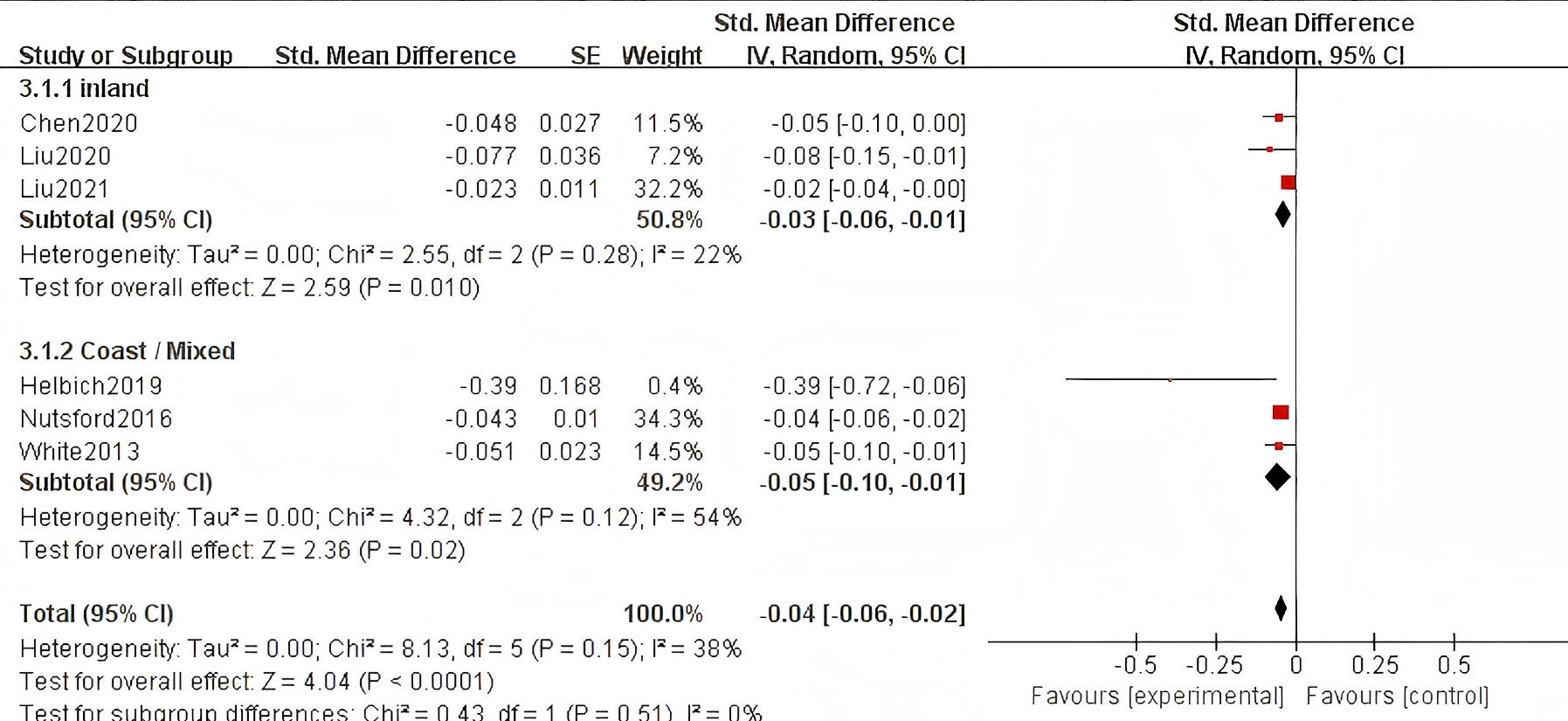

Supplement: Supplementary file 9 [file Image_3.jpeg]
